# Soy Saponins Meditate the Progression of Colon Cancer in Rats by Inhibiting the Activity of **β**-Glucuronidase and the Number of Aberrant Crypt Foci but Not Cyclooxygenase-2 Activity

**DOI:** 10.1155/2013/645817

**Published:** 2013-10-02

**Authors:** Yu-Wei Guo, Yue-Hwa Chen, Wan-Chun Chiu, Hsiang Liao, Shyh-Hsiang Lin

**Affiliations:** School of Nutrition and Health Sciences, Taipei Medical University, 250 Wu-Hsing Street, Taipei 110, Taiwan

## Abstract

*Objective*. The effect of extracted crude soybean saponins on preneoplastic lesions, aberrant crypt foci (ACF), and the related mechanism were investigated. *Research Methods and Procedures*. Rats were assigned into five groups according to different doses of extracted crude soybean saponins and received 1,2-dimethylhydrazine (DMH) injection in week 5. In week 15, all rats were sacrificed. The number of ACFs, the cyclooxygenase-2 (COX-2) protein expression, the level of prostaglandins E2 (PGE2), and the activity of **β**-glucuronidase were examined. *Results*. Results revealed that the consumption of extracted crude soybean saponins decreased the number of ACFs and the activity of **β**-glucuronidase in rats, while the expression of COX-2 protein and PGE2 level were not affected. *Conclusions*. Soybean saponins were effective in inhibiting colon cancer by downregulating the activity of **β**-glucuronidase in colonic mucosa but not the COX-2 protein expression and PGE2 level.

## 1. Introduction

 Dietary habit is one of the major factors that cause colon cancer. It was reported that the colon cancer mortalities are higher in the countries where people consume more animal fat [1]. On the other hand, fiber-rich foods increase the volume of feces and promote the creeping motion of the large intestine, hence, reduce the contacting time of carcinogens in the body [2]. The development of tumor cells the early stage is closely related to inflammatory responses. Immune response factors such as cytokines, reactive oxygen species (ROS), proinflammatory enzyme cyclooxygenase-2 (COX-2), and nitric oxide synthase (iNOS) may accelerate the development of cancer [3]. It has been indicated that colon cancer progression could be slowed down by reducing the expression of COX-2 protein or reducing inflammatory reaction [4, 5]. At the location of inflammation, iNOS may cause the overproduction of nitric oxide, which results in damages on DNA repair and promotes the proliferation of cancer cells [6]. In the early stage of colon cancer in human, lowering the expression of iNOS could be used to reduce the formation of cancer cells [7]. In addition, carcinogens may increase the activities of COX-2 and iNOS on the mucosa of the colon, which also causes the promotion of cancer occurrence [7]. Both COX-2 and iNOS are upregulated by NF-*κ*B. It was indicated that by reducing NF-*κ*B expression, the occurrence of cancer can be reduced [8].

There are several enzymes other than immune response factors, such as nitroreductase, azoreductase, and *β*-glucuronidase produced by the bacteria in the intestine, that are closely related to cancer occurrence. Those enzymes may convert some procarcinogens to carcinogens [9]. Hence, by inhibiting the activities of those enzymes, the chance of cancer formation can also be reduced. 

Soy saponins are amphiphilic compounds and can binnd to the phospholipids and cholesterol with the hydroxyl groups on the aglycone moiety of the compounds [10], hence, change the structure of the cell membrane. Soy saponins have been found to have inhibitory effects *in vitro* on cancer cells [11] by changing the cell morphology, cell proliferation enzymes, and cell growth. In this study, we investigated the effect and mechanism of soy saponins on preventing colon cancer in rats by observing the formation of aberrant crypt foci (ACF) as well as measuring the inflammatory factors, NF-*κ*B, COX-2, and iNOS, and enzyme activities in the large intestine of rats.

## 2. Materials and Methods

### 2.1. Animals and Sample Collection

 Fifty male Fisher 344 rats, six weeks old, were housed in iron cages (2/cage) and kept under the 12/12 hours light-dark conditions of 50% humidity and a temperature of 23°C. Food and water were available* ad libitum*. Animals were fasted overnight and weighed before sacrifice. Rats were assigned into five groups (Table 1). Modified AIN93G was used as the control (basal) diet. From week 0, rats in DS0, DS0.5, and DS1 consumed the diet containing 0%, 0.5%, 1% soy saponin, respectively, for four weeks before receiving dimethylhydrazine (DMH) injections (20 mg/kg-BW, 2 times/week) for the following 6 weeks. Rats in PS0 and PS0.5 groups consumed the basal diet plus 0% and 0.5% of soy saponins, respectively, before receiving saline injections (2 mL/kg BW, 2 times/week) for the following 6 weeks. The blood samples were collected in week 0 and week 15. At the end of week 15, all rats were sacrificed.

### 2.2. Preparation of Soy Saponin

 Soy saponin were extracted by applying and modifying the method previously reported [12]. The purity of crude saponins extracted was examined by HPLC (TSP, Germany) with commercial soy saponins as the standards (Wako, Japan). The HPLC condition was as follows: C18 column (Vercopak, ODS-3, 4.6 × 250 mm); UV absorbance: 190–350 nm; analyzing temperature: 30°C; flow rate: 1 mL/min; gradient solvent system: solvent A, 0.05% trifluoroacetic acid in water and solvent B, acetonitrile. The gradient program was set as 63% A to 52% A in 38 min.

Blood samples were drown from the abdominal cavity vein and collected in tubes containing heparin at the end of the experiment. The liver was purged with phosphate buffer saline (PBS) and stored in formaldehyde, followed by an observation using H & E stain. The rectum and colon samples were collected and washed with PBS and then stored at −80°C. The average length of the rectum (*n* = 4) of each group was recorded. The colon was divided into 3 sections, near cecum section, middle section, and near anus section.

### 2.3. Isoflavones Quantification

Isoflavone standards were prepared using commercially available 25 ppm Daidzin (30408, Fluka), Daidzein (Wako), Genistein (G6649, Sigma) and Genistin (G0897, Sigma). Samples, dissolved in 20 mL 80% methanol (MS1992, Tedia), were prepared from 0.5 g crude soy saponins extract. A high performance liquid chromatography (HPLC) method was used for analysis with C18 column (Inertsil 6, ODS-3, 4.6 × 250 mm, Vercopak). The injection volume was 20 *μ*L. The column temperature was kept constant at 40°C (Super CO-150, Enshine) and the flow rate was 1 mL/min. The UV detection wavelength was set at 248 nm. The binary gradient elution system consisted of two solvents: acetonitrile (A) and acetic acid (0.1% in water, B). Separation was achieved using the following gradient: solvent A, 1–4 min, 15% → 35%; 04–48 min, 35%.

### 2.4. Plasma Isoflavones Level

The isoflavones in the plasma were extracted with tert-butyl methyl ester (TBME) and analyzed with HPLC with C18 column (Inertsil 6, ODS-3, 4.6 × 250 mm, Vercopak) at 40°C. The UV detection wavelength was set at 248 nm and the mobile phase was methanol : ammonium acetate (0.1 M)(40 : 60(pH  4.7)).

### 2.5. Plasma Lipid Analysis

The plasma lipid analysis was performed by using automatic biochemical analyzer (Dri-Chem 3500, Fuji) for total triglyceride (TG) and total cholesterol (TC) determination.

### 2.6. Aberrant Crypt Foci (ACF) Observation

A colon sample was fixed in 10% formalin for 24 hr and flushed with saline on a filter, then dipped in 0.5% methylene blue for 1 min, followed by being washed with distilled water briefly and placed on a microscope slide with the mucosal surface up. The ACFs were classified into: (A) normal crypt, (B) 1 aberrant crypt (AC)/ACF, (C) 2AC/ACF and (D) 3AC/ACF. Then the ACFs containing 1–3 ACs were defined as small ACFs, and those containing more than 3ACs were large ACFs. The numbers of small, large, and total ACFs were determined.

### 2.7. COX-2 Protein Expression in the Colon Mucosa

#### 2.7.1. Protein Determination

Cell protein was extracted and determined using the commercial protein assay kit (BCA1 and B9643, Sigma). Briefly, 200 *μ*L of BCA assay reagent (bicinchoninic acid solution: copper sulfate pentahydrate 4% solution (50 : 1)) was completely mixed with a sample and incubated for 30 min at 37°C. The O.D. was measured at 562 nm.

The COX-2 (MW = 70 kDa) and the iNOS (MW = 130 kDa) protein expressions were measured by using SDS-PAGE with 10% resolving gel and 7% stacking gel followed by western blotting while *β*-actin (42 kDa) was used as an internal control. The primary and secondary antibodies were as follows:

*Primary Antibodies*. *β*-actin (mouse anti-*β*-actin monoclonal antibody, A 5441, SIGMA); COX-2 (goat anti-COX monoclonal antibody, sc-19999, Santa Cruz); iNOS (rabbit anti-NOS2 polyclonal antibody, sc-649, Santa Cruz) diluted in TBST (1 : 1000) which obtained 1% BSA.

*Second Antibodies*. *β*-actin (rabbit anti-mouse IgG, A9044, SIGMA); COX-2 (donkey anti-mouse IgG, sc-2020, Santa Cruz); iNOS (goat anti-rabbit IgG, sc-2004, Santa Cruz).

### 2.8. PGE2 Level in the Colon Mucosa

A colon mucosa sample was homogenized and adjusted to pH 7.2 and then mixed with 2–4 times volume of acetone and incubated for 5 min in room temperature. The sample was centrifuged at 1500 × g for 10 min. The acetone in the supernatant was removed and the PGE2 was determined with commercial kit (Prostaglandin E_2_ EIA Kit, Cayman). The homogenization buffer was prepared by dissolving 13.69 g phosphate in ddH_2_O containing 357.8 mg EDTA and 3.6 mg indomethacin and adding ddH_2_O to 1000 mL (adjust pH to 7.4).

### 2.9. *β*-Glucuronidase Enzyme Activity

A sample of 20 *μ*L and 200 *μ*L GUS (31.5 mg *p*NPG, N-1627, Sigma, dissolved in 100 mL 50 mM Na_3_PO_4_, pH 7.0) were mixed well, followed by performing an enzyme-linked immunosorbent assay (ELISA) at 415 nm every 10 min for an hour. The enzyme activity was acquired according to the Lowry method [13]. 

### 2.10. Liver Biopsy

Liver samples were stained with Hematoxylin and eosin (H & E) dye and subjected to pathological observation.

### 2.11. Statistical Analysis

Data were analyzed with one-way analysis of variance (ANOVA) followed by Fisher's least Significant Differences (LSD) and Dunnett post hoc analysis. All values were expressed as the mean value ± SEM. All group comparisons were considered significant at *P* < 0.05.

## 3. Results

### 3.1. Saponin and Isoflavone Content

One gram of the soy extract contains saponin 778.3 ± 3.8 mg and isoflavone 2.8 ± 0.3 mg, including daidzin 1180 ± 44 *μ*g, genistin 923 ± 11 *μ*g, daidzein 173 ± 58 *μ*g, and genistein 474 ± 197 *μ*g.

### 3.2. Food Intake, Body Weight, and the Organ Weight of the Rats

**Figure 1:**
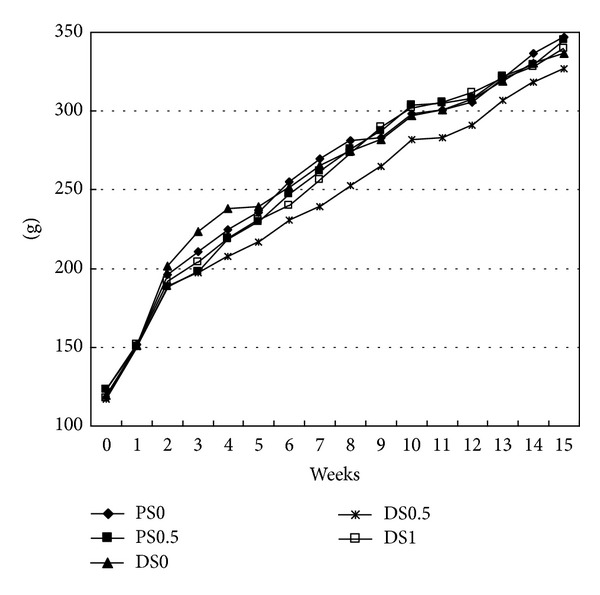
Body weights of rats in the experimental period^1,2^. ^1^PS0: received saline and fed AIN93G diet; PS0.5: received saline and fed AIN93G diet containing 0.5% saponins; DSO: received 1,2-dimethylhydrazine (DMH) and fed AIN93G diet; DS0.5: received DMH and fed AIN93G diet containing 0.5% saponins; DS1: received DMH and fed AIN93G diet containing 1% saponins. ^2^There was no statistic difference between each other (*P* > 0.05).

The average body weight of rats in all groups increased as the experiment proceeded but was not significant between groups. The average change per day in body weight was 1.77 ± 0.04 g in DS0, 1.68 ± 0.06 g in DS0.5, 1.79 ± 0.06 g in DS1, 1.86 ± 0.06 g in PS0, and 1.85 ± 0.08 g in PS0.5. Those receiving DMH injection tended to have a lower body weight increase compared to those receiving saline injection. The average daily food intake of rats was 16.44 ± 0.26 g in DS0, 15.45 ± 0.35 g in DS0.5, 15.95 ± 0.33 g in DS1, 16.78 ± 0.24 g in PS0, and 15.85 ± 0.38 g in PS0.5. There was no significant difference in average daily food intake. The addition of soy extract and injection of DMH did not alter the food consumption (Figure 1). The percentage of the liver weight in body weight was 3.80 ± 0.09% in DS0, 3.77 ± 0.15% in DS0.5, 3.41 ± 0.13% in DS1, 3.79 ± 0.12% in PS0, and 3.58 ± 0.19% in PS0.5, and no statistical difference was found. No significant difference in the length and weight of the colon was detected among groups either.

### 3.3. Aberrant Crypt Foci (ACF) and Cyclooxygenase-2

**Figure 2:**
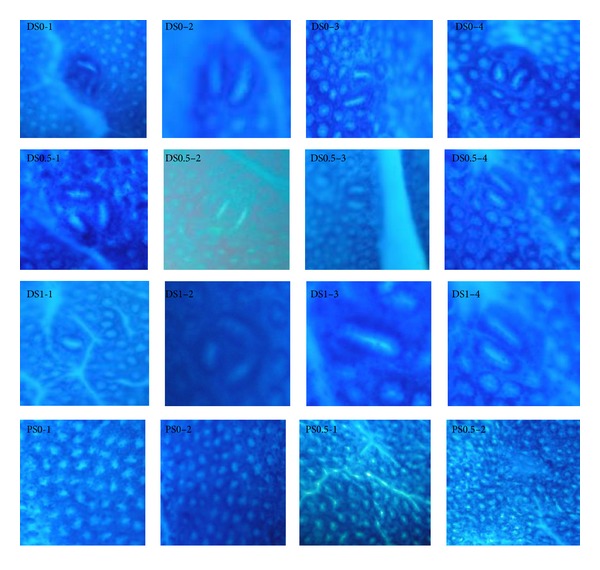
The pathological assessment performed by methylene blue staining in the ACFs of the colonic mucosa of rats^1,2^. ^1^ACFs: aberrant crypt foci. ^2^PS0: received saline and fed AIN93G diet; PS0.5: received saline and fed AIN93G diet containing 0.5% saponins; DSO: received 1,2-dimethylhydrazine (DMH) and fed AIN93G diet; DS0.5: received DMH and fed AIN93G diet containing 0.5% saponins; DS1: received DMH and fed AIN93G diet containing 1% saponins.

ACFs with 1-3 aberrant crypts (ACs) are classified as “small” ACFs, and those with more than 3 AC are “large” ACFs [14]. Our results show that the numbers of small ACFs were negatively correlated to saponin dosages (*P* < 0.05, Table 2). Also, rats in DS1 group had a lower count of large ACFs (>3 ACs) than those in DS0 group did (*P* < 0.05, Table 3). The results also show that there were aberrant crypt foci (ACFs) with 1–6 aberrant crypts (AC) in the DS0, DS0.5, and DS1 groups (Figure 2). No ACFs were found in the PS0 and PS0.5 groups.

 On the other hand, the DS1 group had a lower number of total ACFs in the cecum region than the DS0 group did (*P* < 0.05). In the middle section of the colon, DS1 group had less ACFs than DS0 group and DS0.5 group (*P* < 0.05). At the anal end, the DS0.5 group and the DS1 group had lower total ACFs than the DS0 group (*P* < 0.05). In the whole colon section, the ACFs numbers were negatively correlated to the dose of saponin treated in the experiment (*P* < 0.05) (Table 4). 

**Figure 3:**
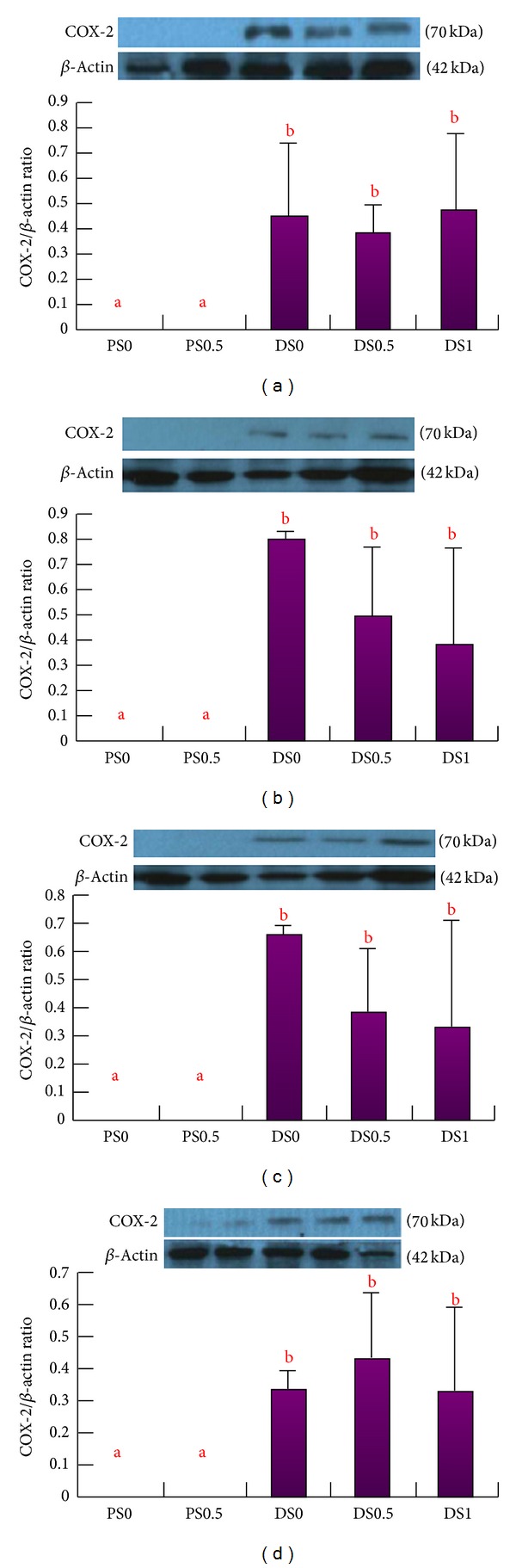
The expression of COX-2 protein in the colonic mucosa^1, 2^. (a) The total expression of COX-2 protein in the colonic mucosa. (b) The expression of COX-2 protein in proximal colonic mucosa. (c) The expression of COX-2 protein in middle colonic mucosa. (d) The expression of COX-2 protein in distal colonic mucosa. ^1^Data are expressed as mean ± SEM (*n* =  3~4). ^2^The image of western blot was representative. ^ab^Letters at the top of each bar sharing the different superscripts are significantly different (*P* < 0.05).

**Figure 4:**
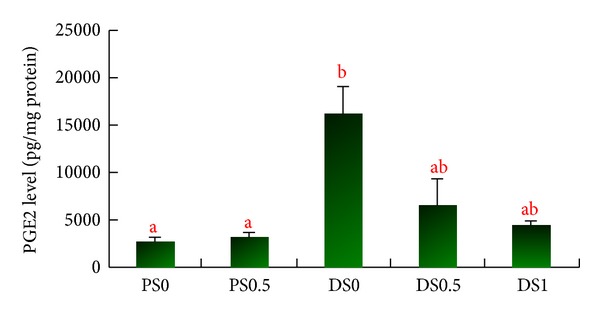
The PGE2 levels in the colonic mucosa^1,2^. ^1^Data are expressed as mean ± SEM (*n* =  3~4). ^2^PS0: received saline and fed AIN93G diet; PS0.5: received saline and fed AIN93G diet containing 0.5% saponins; DSO: received 1,2-dimethylhydrazine (DMH) and fed AIN93G diet; DS0.5: received DMH and fed AIN93G diet containing 0.5% saponins; DS1: received DMH and fed AIN93G diet containing 1% saponins. ^ab^Letters at the top of each bar sharing the different superscripts are significantly different (*P* < 0.05).

 After the injection of DMH, the expressions of COX-2 protein were more significant in the saponin-treated groups (DS0, DS0.5, and DS1) than the PS groups, however, not significant among the DS0, DS0.5, and DS1 groups, and there was no COX-2 expression in the PS groups. There was no significant difference in the colon section regarding the expression of COX-2, while no change in iNOS expression was detected in the experiment (Figures 3(b) and 3(c)). The concentration of PGE2 in DS0 was 16205 ± 2910 pg/mL, 6523 ± 2823 pg/mL in DS0.5, and 4507 ± 396 pg/mL in DS1, while it was 2704 ± 415 pg/mL in PS0 and 3146 ± 537 pg/mL in PS0.5. The level of PGE2 in DS0 was significantly higher than that in PS0 (*P* < 0.05). There was no significant difference among DS0, DS0.5, and DS1 (Figure 4).

**Figure 5:**
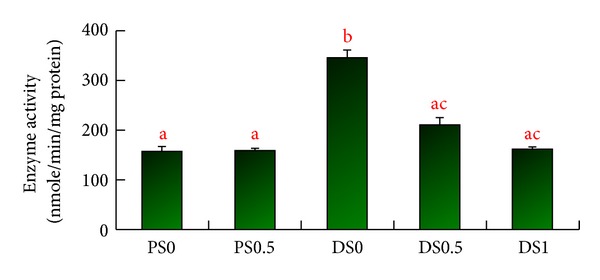
The activities of *β*-glucuronidase in the colonic mucosa^1,2, 3^. ^1^Data are expressed as mean ± SEM (*n* =  3~4). ^2^PS0: received saline and fed AIN93G diet; PS0.5: received saline and fed AIN93G diet containing 0.5% saponins; DSO: received 1,2-dimethylhydrazine (DMH) and fed AIN93G diet; DS0.5: received DMH and fed AIN93G diet containing 0.5% saponins; DS1: received DMH and fed AIN93G diet containing 1% saponins. ^3^The enzyme activities are derived from the changes of activity at 0 and 60 mins. ^abc^Letters at the top of each bar sharing the different superscripts are significantly different (*P* < 0.05).

 The relative activity of *β*-glucuronidase of the colon in DS0 was 346.9 ± 15.6 nmole/min/mg protein, significantly higher than those in PS0 (158.4 ± 8.8 nmole/min/mg protein), DS0.5 (209.6 ± 17.1 nmole/min/mg protein), and DS1 (163.7 ± 5.2 nmole/min/mg protein) (*P* < 0.05) (Figure 5).

## 4. Discussion

Soy compositions have been shown to have anticancer effects, and the saponins are ones of those that are effective [15]. In this experiment, we demonstrated the effect of the soy saponin consumption on preventing the formation of colon cancer. It was indicated that the size and number of aberrant crypt foci (ACFs) increased as the experiment time increased [16]. Also, the number of ACFs can be an indicator for ACFs development [17]. In this experiment, after the injection of DMH, the number of total ACFs was 27.0 ± 1.9, in which the largest one had 6 AC. Thus, with the results of ACFs development, it was applicable to evaluate the effect of saponins on the prevention of colon cancer. The number of ACFs on the colon mucosa is affected by the type, age, and sex of the animal, frequency and dosage of the carcinogen injected, the sections in the colon, and the personnel who is involved in counting ACFs [16]. The mostly used carcinogen for inducing colon cancer in the animal model is azoxymethane (AOM), the metabolite of dimethylhydrazine (DMH) but more toxic than DMH. It was found that the injection of 20 mg/kg wt of DMH for 5 weeks resulted in 28–30 ACFs [18, 19]. The injection of AOM in similar dosages produced much more severe symptoms, in which more than 200 ACFs were induced [20, 21]. In our experiment, rats received 20 mg/kg wt of DMH twice a week for 6 consecutive weeks that resulted in an average of 27 ACFs/rat with 1–6 ACs induced. Thus, using DMH was successful in inducing ACFs and can be used as an alternative method for colon cancer research when using an animal model. In addition to ACFs, the biomarkers for determining the early stages of colon cancer may include mucin-depleted foci (MDF), beta-catenin-accumulated crypts (BCAC), and dysplastic ACF (DACF) [19, 22, 23]. However, ACFs are now used frequently as markers of cancer due to the simplicity in performing without tissue biopsy and the results usually being closely related to the occurrence of tumor [24].

It was shown that soy saponin was effective in inhibiting the growth of the HT-29 human colon cancer cell line by changing the cell morphology and the cell proliferation enzymes [11]. It was indicated that the numbers of total and large aberrant crypt foci (ACF) are reliable indicators of colon cancer occurrence [14]. We found that as the dosage of soy saponin extract in the diet increased, the number of total ACFs decreased. Soy saponins were also found to have cancer-protecting ability by reducing the expression of mRNA and the amount of matrix metalloproteinases (MMP-2 and MMP-9) in HT-1080 cells [25]. It was also shown that triterpenoid B-group soy saponins may induce the autophagic capacity of HTC-15 cells by modulating the activity of extracellular regulated kinase 1/2 (ERK1/2) [26]. A reduced ERK1/2 expression is related to a reduction of ACFs number [27]. However, it was also indicated that the formation of ACFs was more related to a suppressed apoptosis of cancer cells by way of modulating the epidermal growth factor (EGF) signaling pathway rather than the phosphorylated Akt and Erk [28].

 Soy isoflavones, different from saponins that cannot be absorbed by the small intestine, also have been found to reduce the occurrences of several cancers including prostate cancers and breast cancers [29, 30]. A dose of 40 mg/kg BW successfully decreased the tumor burden and tumor size in SD rats [31]. In our experiment, it was due to the fasting time (12 hours) and the short half-life of isoflavone in rats (8.4 hr for genistein and 5.8 hr for daidzein) before the blood was drawn at each time period, that no isoflavone was detected in the blood samples. Also, 1 gram of the soybean saponin extract contained 778.3 mg of saponin and 2.75 mg of isoflavone, including daidzin 1180 ± 44 *μ*g, genistin 923 ± 11 *μ*g, daidzein 173 ± 58 *μ*g, and genistein 474 ± 197 *μ*g. Rats in the group of 1X soy saponin extract (DS1) consumed 0.16 g soy saponin and 0.55 mg isoflavone (daidzin 0.24 mg, genistin 0.17 mg, daidzein 0.03 mg, and genistein 0.09 mg) a day. It was found that 6,7,4′-trihydroxyisoflavone (6,7,4′-THIF), a metabolite of daidzein, inhibited the growth of HCT-116 human colon cancer cells [32]. 6,7,4′-THIF also arrested the cell cycle of HCT-116 at the S and G_2_/M phases and reduced the expression of cyclin-dependent kinase 2 (CDK2). Isoflavone may also inhibit the growth of colon cancer cells, DLD-1, by increasing the expression of estrogen receptor (ER)-*β* [31]. Although the isoflavone in the diet might not be enough to lower the risk of colon cancer, but together with saponins, they might have shown a synergic effect on colon cancer inhibition.

Isoflavones and saponins also have blood cholesterol and blood lipid lowering effects. It was indicated [33] that the total blood cholesterol and triglyceride in C57B1/6J mice were decreased by consuming the high-fat diet containing 0.2% of isoflavone a day. The structure of soy saponins can bind to cholesterol and reduce the absorption of both exogenous and endogenous cholesterol as well as the resorption of bile acid from the gut [34]. However, in our experiment, since the diets were not high-fat diets and the blood lipid level in each group was still in a normal range throughout the experiment period, there was no significant difference in the changes of blood triglyceride and total cholesterol among each group (Table 5).

Cyclooxygenase (COX) and inducible nitric oxides (iNOs) have been used as the index of colon cancer occurrence [35]. Among three types of COX (COX-1-COX-3) only COX-2 gene expression was found to increase in human colon cancer [36]. During a cancer progression stage, COX-2 activity significantly elevates [37]. Inhibition of COX-2 activity may be a preventive method of colon cancer. In this investigation, however, COX-2 and iNOs activities did not change significantly, which may be due to a not-long-enough experiment period or a not sufficient amount of protein produced. In recent years, *β*-glucuronidase activity in the colon has become one of the major indices in the etiology of colon cancer. The enzyme activity increases as the colon cells proliferate abnormally [38]. It was shown that certain types of dietary fiber may have colon cancer preventive effect via changing the secondary bile acids resorption and reducing the activity of bacterial enzymes such as *β*-glucuronidase, which plays a role in carcinogenesis [39]. Rats fed with high-fat diet also resulted in elevated levels of bacterial *β*-glucuronidase activity in the large intestine [40]. It has been found that *β*-glucuronidase activity may be the most important key factor in the genotoxicity of the food-caused colon cancer [41]. In our experiment, the activity of *β*-glucuronidase was significantly increased after the injection of DMH and was reduced by consuming the diet containing soy saponins, which indicates that soy saponins can be one of the ingredients for colon cancer prevention. In conclusion, we found that soy saponins reduced the number of ACFs by reducing the activity of *β*-glucuronidase in colonic mucosa but not the COX-2 protein expression and PGE2 level.

## Figures and Tables

**Table 1:** Diet composition in each group.

(g/kg)	DS0	DS0.5	DS1	PS0	PS0.5
Corn starch	529.5	529.5	529.5	529.5	529.5
Casein	200	200	200	200	200
Sucrose	100	95	90	100	95
Cellulose	50	50	50	50	50
Soybean oil	70	70	70	70	70
Mineral mix^1^	35	35	35	35	35
Vitamin mix^1,2^	10	10	10	10	10
L-cystine	3	3	3	3	3
Choline	2.5	2.5	2.5	2.5	2.5
T-butylhydroquinine	0.014	0.014	0.014	0.014	0.014
Crude saponins	—	5	10	—	5

Energy (kcal/kg)	4147.94	4147.9	4147.94	4147.94	4147.94

^1^The composition of AIN-93 vitamin mixture and AIN-93 mineral mixture. Mixtures are as described in the American Institute of Nutrition [42].

^2^Containing Vitamin A palmitate 1.6 g/kg.

**Table 2:** The counts of small ACFs in rats^1,2^.

	The incidence of ACFs^3^	1AC	2AC	3AC	Small^4^
PS0	0/4	ND^5^	ND	ND	ND
PS0.5	0/4	ND	ND	ND	ND
DS0	6/6	10.16 ± 1.22^a^	9.00 ± 1.46^a^	5.83 ± 0.70	25.00 ± 1.89^a^
DS0.5	6/6	5.67 ± 0.59^ab^	6.00 ± 0.75^ab^	3.83 ± 0.41	15.50 ± 0.54^b^
DS1	6/6	2.83 ± 0.39^b^	1.83 ± 0.50^b^	3.00 ± 0.63	7.67 ± 1.32^c^

^1^Data are expressed as mean ± SEM (*n* = 6).

^2^PS0: received saline and fed AIN93G diet; PS0.5: received saline and fed AIN93G diet containing 0.5% saponins; DS0: received 1,2-dimethylhydrazine (DMH) and fed AIN93G diet; DS0.5: received DMH and fed AIN93G diet containing 0.5% saponins; DS1: received DMH and fed AIN93G diet containing 1% saponins.

^3^The incidence of ACFs is the number of ACF rats/the number of total rats.

^4^Small ACFs: 1~3AC/ACF.

^5^ND: No detection.

^abc^Letters in each column sharing the different superscripts are significantly different (*P* < 0.05).

**Table 3:** The counts of large ACFs in rats^1,2^.

	The incidence of ACFs^3^	4AC	5AC	6AC	Large^4^
PS0	0/4	ND^5^	ND	ND	ND
PS0.5	0/4	ND	ND	ND	ND
DS0	6/6	1.67 ± 0.27	0.17 ± 0.09	0.17 ± 0.09	2.00 ± 0.20^a^
DS0.5	6/6	1.17 ± 0.17	ND	ND	1.17 ± 0.17^ab^
DS1	6/6	0.17 ± 0.09	0.17 ± 0.09	ND	0.33 ± 0.12^b^

^1^Data are expressed as mean ± SEM (*n* = 6).

^2^PS0: received saline and fed AIN93G diet; PS0.5: received saline and fed AIN93G diet containing 0.5% saponins; DS0: received 1,2-dimethylhydrazine (DMH) and fed AIN93G diet; DS0.5: received DMH and fed AIN93G diet containing 0.5% saponins; DS1: received DMH and fed AIN93G diet containing 1% saponins.

^3^The incidence of ACFs is the number of ACF rats/the number of total rats.

^4^Large ACFs: >3AC/ACF.

^5^ND: No detection.

^ab^Letters in each column sharing the different superscripts are significantly different (*P* < 0.05).

**Table 4:** The distribution of ACFs according to different sections of the colon^1,2^.

	The incidence of ACFs^3^	Proximal	Middle	Distal	Total
PS0	0/4	ND^4^	ND	ND	ND
PS0.5	0/4	ND	ND	ND	ND
DS0	6/6	4.50 ± 0.67^a^	7.17 ± 0.62^a^	15.50 ± 1.35^a^	27.00 ± 1.91^a^
DS0.5	6/6	2.00 ± 0.28^ab^	4.83 ± 0.33^a^	9.83 ± 0.43^b^	16.67 ± 0.44^b^
DS1	6/6	1.00 ± 0.35^b^	1.83 ± 0.50^b^	5.17 ± 0.81^b^	8.00 ± 1.41^c^

^1^Data are expressed as Mean ± SEM (*n* = 6).

^2^PS0: received saline and fed AIN93G diet; PS0.5: received saline and fed AIN93G diet containing 0.5% saponins; DS0: received 1,2-dimethylhydrazine (DMH) and fed AIN93G diet; DS0.5: received DMH and fed AIN93G diet containing 0.5% saponins; DS1: received DMH and fed AIN93G diet containing 1% saponins.

^3^The incidence of ACFs is the number of ACF rats/the number of total rats.

^4^ND: No detection.

^abc^Letters in each column sharing the different superscripts are significantly different (*P* < 0.05).

**Table 5:** The change of blood lipid in rats from week 0 to week 15^1,2^.

Group	TG (mg/dL)			TC (mg/dL)		
Week	0	15	Δ0~15*	0	15	Δ0~15*
PS0	102.6 ± 36.6	111.4 ± 36.4	8.75 ± 2.9	75.9 ± 15.7	64.5 ± 9.6	−11.4 ± 4.1
PS0.5	140.0 ± 51.7	169.5 ± 73.4	29.5 ± 19.6	71.9 ± 10.5	75.0 ± 12.8	3.4.4
DS0	150.6 ± 43.7	122.4 ± 29.8	−28.3 ± 9.4	71.7 ± 12.1	63.9 ± 9.7	−7.8 ± 3.5
DS0.5	111.7 ± 40.2	90.2 ± 18.3	−21.4 ± 7.4	77.3 ± 15.6	53.9 ± 20.9	−23.4 ± 5.0
DS1	126.6 ± 67.4	91.6 ± 25.6	−35.0 ± 11.6	77.0 ± 15.8	61.3 ± 10.4	−8.0 ± 7.7

^1^Data are expressed as mean ± SEM (*n* = 8~10).

^2^PS0: received saline and fed AIN93G diet; PS0.5: received saline and fed AIN93G diet containing 0.5% saponins; DSO: received 1,2-dimethylhydrazine (DMH) and fed AIN93G diet; DS0.5: received DMH and fed AIN93G diet containing 0.5% saponins; DS1: received DMH and fed AIN93G diet containing 1% saponins.

*There was no statistic difference between each other (*P* > 0.05).
